# Chinese text classification by combining Chinese-BERTology-wwm and GCN

**DOI:** 10.7717/peerj-cs.1544

**Published:** 2023-08-17

**Authors:** Xue Xu, Yu Chang, Jianye An, Yongqiang Du

**Affiliations:** College of Science, Tianjin University of Commerce, Tianjin, China

**Keywords:** Chinese text classification, GCN, Chinese-BRRTology-wwm, Pointwise mutual information, Loss function, Transductive learning

## Abstract

Text classification is an important and classic application in natural language processing (NLP). Recent studies have shown that graph neural networks (GNNs) are effective in tasks with rich structural relationships and serve as effective transductive learning approaches. Text representation learning methods based on large-scale pretraining can learn implicit but rich semantic information from text. However, few studies have comprehensively utilized the contextual semantic and structural information for Chinese text classification. Moreover, the existing GNN methods for text classification did not consider the applicability of their graph construction methods to long or short texts. In this work, we propose Chinese-BERTology-wwm-GCN, a framework that combines Chinese bidirectional encoder representations from transformers (BERT) series models with whole word masking (Chinese-BERTology-wwm) and the graph convolutional network (GCN) for Chinese text classification. When building text graph, we use documents and words as nodes to construct a heterogeneous graph for the entire *corpus*. Specifically, we use the term frequency-inverse document frequency (TF-IDF) to construct the word-document edge weights. For long text corpora, we propose an improved pointwise mutual information (PMI*) measure for words according to their word co-occurrence distances to represent the weights of word-word edges. For short text corpora, the co-occurrence information between words is often limited. Therefore, we utilize cosine similarity to represent the word-word edge weights. During the training stage, we effectively combine the cross-entropy and hinge losses and use them to jointly train Chinese-BERTology-wwm and GCN. Experiments show that our proposed framework significantly outperforms the baselines on three Chinese benchmark datasets and achieves good performance even with few labeled training sets.

## Introduction

Text classification is a fundamental task in natural language processing (NLP) and has been widely used in information retrieval, spam detection, sentiment analysis and other fields (Wang, 2010; Cambria et al., 2013; Ullah, Khan & Nawi, 2022).

Text representation is an indispensable intermediate step for text classification. Traditional methods represent text with handcrafted features, such as bag-of-words (Mccallum & Nigam, 1998) and N-grams (Lin & Hovy, 2003), and then adopt machine learning algorithms for text classification. However, these methods do not consider contextual information. With the introduction of distributed word vector representations, neural network-based methods have substantially improved the performance of text classification by encoding text semantics. These methods use word2vec, global vectors (GloVe) (da Costa, Oliveira & Fileto, 2023), *etc*., to represent text as the semantic information of words and then adopt deep learning models such as convolutional neural networks (CNNs) (Kim, 2014; Zhai et al., 2023) and recurrent neural networks (RNNs) (Mousa & Schuller, 2017; Liu & Song, 2022) for text classification. CNNs and RNNs prioritize locality and sequentiality; therefore, they can effectively capture semantic and syntactic information in locally consecutive word sequences but may ignore global word co-occurrence in corpora with nonconsecutive and long-distance semantics.

Transductive learning (Vapnik, 1998) is a text classification method that uses both labeled and unlabeled examples during the training process. GNNs serve as effective approaches for transductive learning. Several studies have used GNNs for text classification, such as TextGCN (Yao, Mao & Luo, 2019), BertGCN (Lin et al., 2021) and HGAT (Yang et al., 2021). In these works, the relations between documents and words are modeled by finding the underlying graph structure information in text data to construct graphs. For example, the nodes in a graph represent textual units, such as documents and words, and the edges are constructed based on the co-occurrence relations between the graph nodes. The application of GNNs can transform text classification problems into graph or graph node classification problems. GNNs are effective in tasks with rich relational structures and can preserve global graph structure information.

Based on the above analysis, the existing text classification methods have some limitations with respect to text feature extraction. First, some methods (Mousa & Schuller, 2017; Liu & Song, 2022) use RNNs and long short-term memory (LSTM) to process serialized data, ignoring the global structure information contained in the given text. Second, TextGCN (Yao, Mao & Luo, 2019) effectively obtains text structure information but ignores contextual semantic information. BertGCN (Lin et al., 2021) combines text structure information and semantic information. However, the above works all addressed English text classification issues, and their applications in Chinese text classification need to be discussed and verified. Moreover, none of these studies considered the applicability of their graph construction methods to long or short texts, nor did they take into account the distance between co-occurring words when calculating PMI. Incorporating such factors would enable better information propagation between nodes, thus enhancing the accuracy of text classification.

Inspired by the TextGCN (Yao, Mao & Luo, 2019) and BertGCN (Lin et al., 2021) for English text classification, we use documents and words as nodes to construct a heterogeneous graph for the entire *corpus* and propose Chinese-BERTology-wwm-GCN, a framework that combines the advantages of both large-scale pretraining and transductive learning for Chinese text classification. However, different from the above work, our contributions are as follows.

(1) We focus on the problem of Chinese text classification and use Chinese-BERTology-wwm to obtain the text representation, which is pretrained based on whole word masking. Furthermore, we apply Chinese-BERTology-wwm to fine-tune the downstream tasks. By jointly training the Chinese-BERTology-wwm and GCN modules for Chinese text classification, the proposed framework can leverage the advantages of both components.

(2) When building text graphs, we use different word-word edge weights for long and short text corpora respectively.

For long text corpora, we propose an improved pointwise mutual information ( $PM{I^*}$) measure for words to represent the edge weights between words. The number of co-occurrences is influenced by the length of the sliding window, and words that are closer together tend to have stronger relationships. In order to calculate the PMI of words more accurately, we consider the distances between co-occurring words when calculating their co-occurrences.

For short text corpora, the co-occurrence information between words is often limited due to the small number of words in each document. Therefore, we use Chinese-BERTology-wwm to obtain the representation of words and use cosine similarity to represent the word-word edge weights. This can enrich the graph-structured relationships of short texts and enhance the propagation of information between nodes.

(3) During the training stage, we integrate the cross-entropy and hinge losses for the joint training of Chinese-BERTology-wwm and GCN. The cross-entropy loss has a higher learning rate, as it indicates the distance between the predicted and actual probability distributions of classes. However, it only depends on the logarithm of the probability that the model predicts the correct class. On the other hand, the hinge loss function demands not only correct classification but also that the loss will be 0 when the certainty is sufficiently high. Therefore, the hinge loss function imposes stronger constraints on the model and requires a higher level of learning. In this study, we take full advantage of the cross-entropy and hinge losses and effectively integrate them for the joint training of Chinese-BERTology-wwm and GCN.

For the Chinese text classification task, we conduct extensive empirical studies to examine the performance of our proposed framework and the baselines on some Chinese benchmark datasets with careful analyses. The experimental results show that our proposed framework can leverage the advantages of both Chinese-BERTology-wwm and GCN modules by jointly training them and achieves good performance even with few labeled training sets. Furthermore, improving the construction of text graph and integrating the two loss functions into the training process of Chinese-BERTology-wwm-GCN can further improve its performance in Chinese text classification.

## Related work

### Chinese text classification

English words are separated by spaces, and words are the smallest language units that can be used independently. Compared with English words, Chinese words are composed of one or more characters, and there is no clear definition between words in the Chinese language. Therefore, words are the basic semantic units, and word segmentation is the basic link of traditional Chinese NLP, which has an important impact on the subsequent text classification accuracy (Huang & Zhao, 2007). Chinese text classification methods include machine learning methods, deep learning methods, GNNs methods and large-scale pretrained models.

### Machine learning-based methods

Traditional machine learning methods adopt handcrafted features such as bag-of-words (Mccallum & Nigam, 1998), N-grams (Lin & Hovy, 2003) and TF-IDF (Zhang, Yoshida & Tang, 2009) features as inputs and utilize machine learning algorithms such as support vector machines (SVM) (Joachims, 1998), logistic regression (Genkin, Lewis & Madigan, 2007) and naive Bayes (NB) classifiers (Mccallum & Nigam, 1998) for classification purposes. However, these methods usually rely heavily on complex feature engineering and ignore the word order and semantic information contained in the text, which are important for understanding text semantics. In addition, feature engineering is performed manually. Therefore, these methods have difficulty processing large-scale data, and they cannot solve the highly sparse feature vector problem.

### Deep learning-based methods

Recently, incorporating external knowledge into deep learning to expand text information has become a hot research topic in NLP tasks. Deep learning-based methods usually use word2vec and GloVe (da Costa, Oliveira & Fileto, 2023) to represent text, which can capture the semantic information of words, and use CNNs (Kim, 2014; Zhai et al., 2023), RNNs (Mousa & Schuller, 2017; Liu & Song, 2022) and other deep neural networks (Zhao et al., 2018) for text classification. Compared with machine learning methods, the text feature extraction of deep learning is integrated into the model training process and can effectively encode word orders and semantic information. Deep learning-based methods have substantially improved the performance of text classification.

The representative methods are TextCNN (Kim, 2014), TextRNN (Liu, Qiu & Huang, 2016), LSTM (Mousa & Schuller, 2017) and the gated recurrent unit (GRU) (Chung et al., 2014). CNNs and RNNs prioritize locality and sequentiality but may ignore global word co-occurrences in corpora with nonconsecutive and long-distance semantics. Recently, some other deep learning-based methods have been proposed, such as capsule networks (Wang et al., 2022), attention mechanisms (Chen et al., 2022; Huang et al., 2023) and GNNs (Kipf & Welling, 2016; Veličković et al., 2017; Cao & Kipf, 2018).

### GNN-based methods

Due to the ubiquity of graph structures, research on extending deep learning to graph structures has received increasing attention. As a representative method that combines deep learning with graph data, the emergence of GCN has led to a large class of methods that apply neural network technology to graph data learning tasks, and GNNs have emerged for appropriate tasks (Wu et al., 2020). GNNs are a class of connectivity models that capture the dependencies between graph nodes through information transfer between them. Representative GNNs include GCNs, graph attention networks, graph autoencoding networks, graph generation networks, and graph spatiotemporal networks (Kipf & Welling, 2016; Veličković et al., 2017; Cao & Kipf, 2018). Recent studies (Yao, Mao & Luo, 2019; Lin et al., 2021) have shown that GNNs are effective in tasks with rich structural relationships and can preserve global graph structure information. GNNs serve as effective approaches for transductive learning, which jointly learns representations for both training data and unlabeled test data by propagating label influence through graph edges. Therefore, unlabeled samples also contribute to the representation learning process, enabling strong classification performance even with few labeled documents.

The object of NLP is usually text. Although there are no obvious graph data in text, rich graph structures are hidden in text. The popularity of GNNs has inspired many researches projects in the field of text classification, such as TextGCN (Yao, Mao & Luo, 2019), BertGCN (Lin et al., 2021) and BEGNN (Yang & Cui, 2021), which have demonstrated their effectiveness over traditional statistical feature-based methods.

GCN extracts features from graph data by defining convolutions in a non-Euclidean space. However, the computational complexity of obtaining explicit eigenvalues through matrix eigendecomposition is relatively high. Kipf & Welling (2016) and Defferrard, Bresson & Vandergheynst (2016) used Chebyshev polynomials to fit the convolution kernel and solved the computational complexity problem by parameterizing the convolution kernel. Yao, Mao & Luo (2019) applied GCN to text classification tasks for the first time and proposed TextGCN, which represents documents and words as nodes and constructs a heterogeneous graph over the dataset. TextGCN (Yao, Mao & Luo, 2019) effectively captures the structural information of text, but to some extent, it neglects the semantic and contextual information of text. To solve these problems, some scholars have made improvements by introducing text semantics, syntax and context information (Liu et al., 2020; Yang et al., 2021; Lin et al., 2021). Liu et al. (2020) proposed TensorGCN, which builds text graphs based on semantics, syntax and sequences and effectively integrates heterogeneous information from multiple graphs through intragraph and intergraph propagation strategies. Yang et al. (2021) proposed HGAT, which introduces external semantic information such as entities and topics and learns the relationships between them to alleviate the problem of sparse features in short text. Lin et al. (2021) proposed BertGCN, which follows the text graph of TextGCN and uses the BERT model to represent document nodes. Compared with TextGCN, BertGCN achieves better classification results. However, none of these studies considered the applicability of their graph construction methods to long or short texts.

The above studies are based on the whole *corpus* to build text graph. When the *corpus* is large, it consumes considerable computer memory. Therefore, some studies constructed text graphs based on single documents, thus transforming text classification tasks into graph classification tasks (Huang et al., 2019; Yang & Cui, 2021). Huang et al. (2019) proposed a new GNN-based model that builds graphs for each input text with global parameter sharing instead of a single graph for the whole *corpus*. Yang & Cui (2021) proposed BEGNN, which builds text graphs based on single documents. It uses GNN to extract text features and BERT to extract semantic features, and combines these two types of features at different granularity levels to obtain a more effective representation. TextFCG (Wang et al., 2023) constructs a single graph for all words in each text, labels the edges by fusing the various contextual relations, and uses GNN and GRU for text classification.

### Large-scale pretrained language model-based methods

More recently, the successful proposal of large-scale pretrained models stimulated great interest in applying large-scale pretrained frameworks to text classification (Devlin et al., 2018). As the most representative model, BERT (Devlin et al., 2018) is built on top of the transformer architecture and is pretrained on a large-scale unlabeled text *corpus* in the manner of a masked language model and next-sentence prediction. BERT completes tasks such as text classification, reading comprehension, and sequence annotation by performing pretraining and fine-tuning. Researchers have made great and rapid progress in optimizing BERT-series models (BERTology) based on the original BERT model, such as ERNIE, RoBERTa and ALBERT (Sun et al., 2019; Liu et al., 2019; Lan et al., 2019).

Although various pretrained language models have been released, most of them are based on the English language, and few efforts have been focused on building powerful pretrained language models in other languages. As Chinese and English are among the most widely spoken languages in the world, in the Chinese BERT-based model officially released by Google, Chinese text is segmented according to the granularity of characters, and subwords represent independent Chinese characters. However, traditional Chinese NLP tasks are mostly word-based problems. Recently, Cui et al. (2021) proposed the whole word masking (wwm) strategy for Chinese BERT-series models, where all characters within a Chinese word are masked altogether.

As large-scale pretrained language models, BERT-series models can learn implicit but rich context semantic information from language at scale, and they have the potential to benefit transductive learning. Few studies have made full use of contextual, semantic, and structural information for Chinese text classification. Existing GNN methods (Yao, Mao & Luo, 2019; Liu et al., 2020) all concern English text classification tasks, and their applications in Chinese text classification tasks need to be discussed and verified. Moreover, none of these studies considered the applicability of their graph construction methods to long or short texts, nor did they consider the distances between words when calculating PMI. Incorporating such factors would enable better information propagation between nodes, thus enhancing the accuracy of text classification.

In this study, we make full use of the semantic and structural information of text to propose a Chinese text classification framework that combines Chinese-BERTology-wwm and GCN and improve the construction of text graph for both long and short text corpora.

## Methods

**Figure 1:**
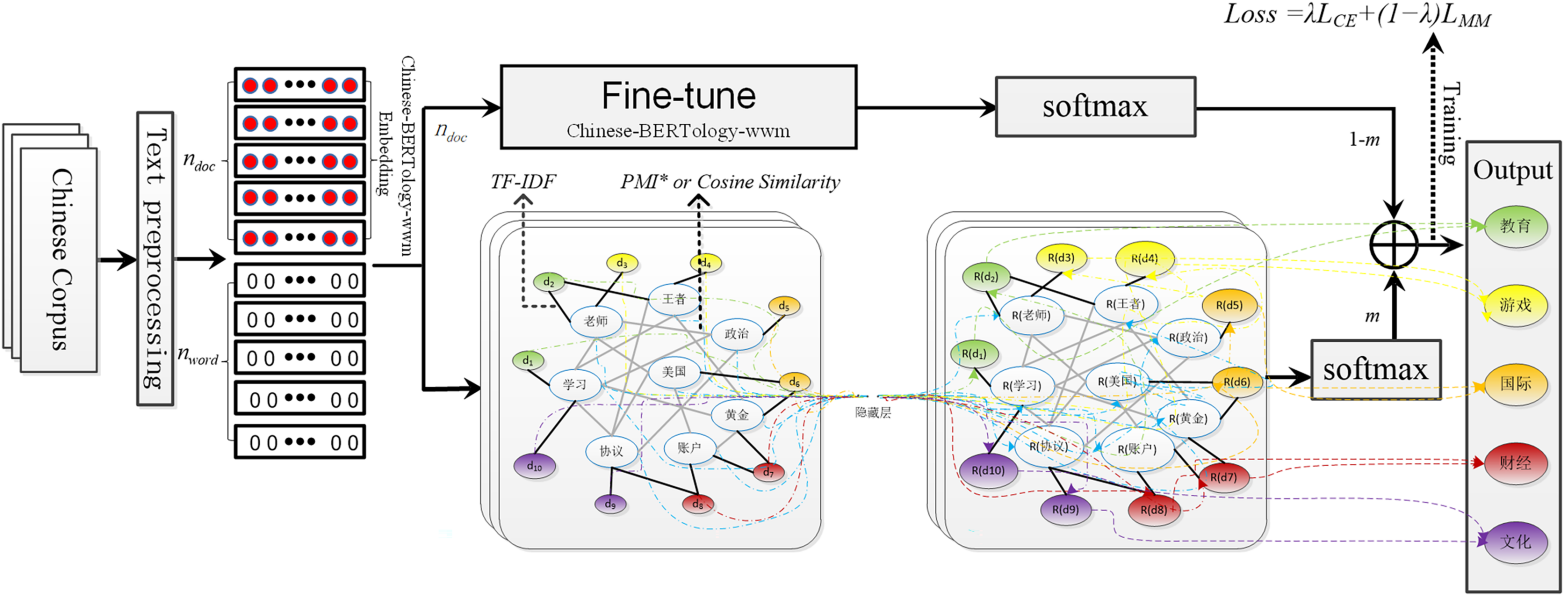
The architecture of Chinese-BERTology-wwm-GCN includes Chinese-BERTology-wwm and GCN modules.

In this section, we present the proposed architecture based on Chinese-BERTology-wwm (Devlin et al., 2018; Cui et al., 2021) and GCN (Kipf & Welling, 2016). The architecture of Chinese-BERTology-wwm-GCN includes Chinese-BERTology-wwm and GCN modules, as shown in Fig. 1. Among them, the input text graph is visualized by extracting partial data from the Toutiao-S dataset.

### Chinese-BERTology-wwm-GCN

GCN (Kipf & Welling, 2016) is a multilayer neural network that operates directly on a graph and induces embedding vectors for nodes based on the properties of their neighborhoods. Therefore, we must first construct the text graph before using GCN for text classification. Formally, consider a graph $G = \left( {V,E} \right)$, where $V$ and $E$ are sets of nodes and edges, respectively. Every node is assumed to be connected to itself. A heterogeneous graph containing word nodes and document nodes is constructed based on the relations between words and documents. Unlike TextGCN (Yao, Mao & Luo, 2019) and BertGCN (Lin et al., 2021), we use different word-word edge weights for long and short text corpora respectively. For long text corpora, we propose an improved pointwise mutual information (PMI*) measure for words according to their word co-occurrence distances to represent the weights of word-word edges. For short text corpora, the co-occurrence information between words is often limited due to the small number of words in each document. Therefore, we use Chinese-BERTology-wwm to obtain the representation of words and use cosine similarity to represent the word-word edge weights. This can enrich the graph-structured relationships of short texts and enhance the propagation of information between nodes. We introduce an adjacency matrix $A$ of $G$, and the connections between words or documents are defined as:

(1) $${A_{ij}} = \left\{ {\matrix{
   {PM{I^*}\left( {i,j} \right),} & {i,j\,are\,words,PM{I^*}\left( {i,j} \right){\mkern 1mu} {\mkern 1mu}  > 0,{\mkern 1mu} \,long\,text\,corpus}  \cr 
   {Cosine\,Similarity\left( {i,j} \right),\;} & {i,\,\,j\,{\mkern 1mu} are{\mkern 1mu} \,words,Cosine\,Similarity\left( {i,j} \right) > {\mkern 1mu} C,{\mkern 1mu} \,short\,text\,corpus}  \cr 
   {TF - ID{F_{ij}},} & {i{\mkern 1mu} \,is{\mkern 1mu} \,document,j{\mkern 1mu} \,is{\mkern 1mu} \,word}  \cr 
   {1,} & {i = j}  \cr 
   {0,} & {otherwise}  \cr 

 } } \right.$$

The diagonal elements of $A$ are set to 1 because of self-loops.

Let the input of the GCN model be $X$, a matrix containing all nodes with their features. In TextGCN (Yao, Mao & Luo, 2019), an identity matrix $X = {I_{{n_{doc}} + {n_{word}}}}$ is used as the initial node feature, where ${n_{doc}}$ represents the number of document nodes and ${n_{word}}$ represents the number of word nodes. In BertGCN (Lin et al., 2021), BERT-series models are used to obtain document embeddings, and the embeddings are used as input representations for the document nodes. The document node embeddings are denoted as ${X_{doc}} \in {R^{{n_{doc}} \times d}}$, where $d$ represents the embedding dimensionality. Due to the particularity of Chinese text, words are the smallest language units that are used independently in Chinese characters, and words can better express the basic information contained in Chinese texts. Therefore, in this work, we adopt Chinese-BERTology-wwm, which is based on whole word masking, to obtain the initial representations of documents and use these representations as the GCN inputs. The initial node feature matrix is derived from the following formula:

(2) $$X = {\left( {\matrix{ {{X_{doc}}} \cr 0 \cr } } \right)_{\left( {{n_{doc}} + {n_{word}}} \right) \times d}}$$

After building the text graph, $X$ is fed into the GCN model, which iteratively propagates the information between the training and test samples. GCN captures the high-order neighborhood information of the nodes through multilayer graph convolution, and the $i$-th layer of the GCN is defined as:

(3) $${L^{\left( i \right)}} = \sigma \left( {\tilde A{L^{\left( {i - 1} \right)\; }}{W^{\left( i \right)}}} \right)$$

where ${L^{\left( {i - 1} \right)}}$ is the $\left( {i - 1} \right)$-th layer of the GCN, $\sigma \left( \cdot \right)$ is the activation function, $\tilde A = {D^{ - {1 \over 2}}}A{D^{ - {1 \over 2}}}$ is the normalized adjacency matrix, $\; D$ is the degree matrix of $G$, and ${W^{\left( i \right)}} \in {R^{{d_{i - 1}} \times {d_i}}}$ is the weight matrix of the $i$-th layer.

We choose ELU() as the activation function, which converges faster than the ReLU() function, and its definition is shown in Eq. (4).

(4) $$\sigma \left( x \right) = \left\{ {\matrix{
   {x,} & {x > {\mkern 1mu} 0}  \cr 
   {\alpha \left( {{e^x} - 1} \right),} & {otherwise}  \cr 

 } } \right.$$

In our preliminary experiment, we found that a two-layer GCN performed better than a one-layer GCN, while more layers did not yield improved performance. This is similar to the results of TextGCN (Yao, Mao & Luo, 2019). ${L^{\left( 0 \right)}} = X$ is the input feature matrix of the model. The output of the two-layer GCN is the final representation of the document node, which is then sent to the softmax layer for classification. The output is shown in Eq. (5).

(5) $${f_{GCN}} = softmax\left( {\tilde A\sigma \left( {\tilde AX{W^{\left( 0 \right)}}} \right){W^{\left( 1 \right)}}} \right)$$

A fully connected output layer is added after Chinese-BERTology-wwm, and $X$ is sent as the input of the softmax layer for classification. The classification output representation of Chinese-BERTology-wwm is shown in Eq. (6), where $W$ is the weight of the fully connected layer.

(6) $${f_{BERT}} = softmax\left( {WX} \right)$$

In this work, we use the Chinese-BERTology-wwm classification function to assist the GCN with text classification. Chinese-BERTology-wwm and the two-layer GCN model are jointly trained to obtain the final classification results.

(7) $$f = m{f_{GCN}} + \left( {1 - m} \right){f_{BERT}}$$

where $0 \le m \le 1$. $m = 1$ represents that the GCN is used for training and prediction; $m = 0$ indicates that the Chinese-BERTology-wwm is used for training and prediction; and $0 < m < 1$ indicates that the GCN and Chinese-BERTology-wwm modules are used for joint training and prediction.

### Improved PMI of words

To calculate the PMI of words, we define a sliding window and use $window\_size$ to represent the length of the sliding window. When the document length is less than $window\_size$, the $window\_size$ value is updated to the document length. $Num$ is the total number of sliding windows in a *corpus*, and $Num\left( i \right)$ is the number of sliding windows in a *corpus* that contain word $i$. In TextGCN (Yao, Mao & Luo, 2019) and BertGCN (Lin et al., 2021), $p\left( {i,j} \right) = Num\left( {i,j} \right)/Num$, and $Num\left( {i,j} \right)$ is the number of sliding windows that contain both word $i$ and $j$. This method does not take into account the distance between co-occurring words. It treats all pairs of words that appear within a sliding window equally and increments the co-occurrence count by one for each pair. However, the number of co-occurrences is influenced by the length of the sliding window, and words that are closer together tend to have stronger relationships. In order to calculate the PMI of words more accurately, we consider the distances between co-occurring words when calculating their co-occurrences. The specific calculation formulas are shown in Eqs. (8)–(11). ${D_t}\left( {i,j} \right)$ is the distance between word $i$ and word $j$ at the $t$-th co-occurrence, and $0 \le {D_t}\left( {i,j} \right) \le window\_t - 1$. $window\_t$ is the length of the window in which word $i$ and word $j$ co-occur at the $t$-th time. Specifically, the improved PMI of words ( $PM{I^*}$) is defined as:

(8) $$PM{I^*}\left( {i,j} \right) = log\displaystyle{{p\left( {i,j} \right)} \over {p\left( i \right)p\left( j \right)}}$$

(9) $$p\left( i \right) = \displaystyle{{Num\left( i \right)} \over {Num}}$$

(10) $$p\left( {i,j} \right) = \displaystyle{{Nu{m^*}\left( {i,j} \right)} \over {Num}}$$

(11) $$Nu{m^*}\left( {i,j} \right) = \mathop \sum \limits_t 1 - \displaystyle{{{D_t}\left( {i,j} \right)} \over {window\_t}}$$

### Loss function integration

When addressing with classification problems, the cross-entropy loss is a commonly used loss function and is defined as:

(12) $${L_{CE}} = - \displaystyle{1 \over N}\mathop \sum \limits_{n = 1}^N \mathop \sum \limits_{k = 1}^K y_n^{\left( k \right)}\;log\hat y_n^{\left( k \right)} = - \displaystyle{1 \over N}\mathop \sum \limits_{n = 1}^N log\hat y_n^{\left( c \right)}$$

The number of documents in a *corpus* is represented by $N$, which is the sample size, and $K$ is the number of sample categories. $y_n^{\left( k \right)}$ represents the actual output result of the $n$-th sample from the $k$-th class, the correct class output is 1, and the other class output is 0. $\hat y_n^{\left( k \right)}$ represents the probability that the model predicts the $n$-th sample to belong to the $k$-th class. $\hat y_n^{\left( c \right)}$ represents the probability that the model predicts that the $n$-th sample belongs to the correct class $c$. Therefore, the cross-entropy loss only depends on the logarithm of the probability predicted by the model for the correct class.

For multiclass classification tasks, the hinge loss function can also be used as the loss function when constructing the objective function, and its definition is as follows:

(13) $${L_{MM}} = \displaystyle{1 \over N}\mathop \sum \limits_{n = 1}^N \mathop \sum \limits_{k = 1}^K max{\left( {0,margin - \hat y_n^{\left( c \right)} + \hat y_n^{\left( k \right)}} \right)^p}$$

In Eq. (13), the value of $p$ is 1 or 2. In this article, the default value of $p$ is 1, and the default value of $margin$ is 1.

From the definition of the hinge loss function, it can be seen that the hinge loss function requires accurate classification and sets the loss value to 0 when the certainty is sufficiently high. Due to this condition, the hinge loss function has higher learning requirements than other loss functions. In contrast, the cross-entropy loss function belongs to the negative log-likelihood family of losses. The gradient of the loss function is large when the model error is high, and learning is faster; conversely, the gradient is small when the model error is low, and learning is slower. Therefore, the cross-entropy loss has a higher learning rate, but it depends only on the logarithm of the probability that the model predicts the correct class.

In this work, we combine the cross-entropy loss function and the hinge loss function and use the loss on the labeled document nodes to jointly optimize the parameters of the Chinese-BERTology-wwm and GCN modules. When training the model, the loss function is defined as:

(14) $${L^*} = \lambda {L_{CE}} + \left( {1 - \lambda } \right){L_{MM}}$$

where $0 \le \lambda \le 1$.

## Experimental details

We perform extensive experiments to verify the effect of the proposed Chinese text classification framework and conduct ablation experiments to demonstrate the role of each module.

### Baseline models

In this article, TextGCN (Yao, Mao & Luo, 2019), Chinese-BERT-wwm (Cui et al., 2021), Chinese-RoBERTa-wwm (Cui et al., 2021), BERT (Devlin et al., 2018) and RoBERTa (Liu et al., 2019) are used as baseline models for comparative experiments.

For the BERTology models (BERT, RoBERTa, Chinese-BERT-wwm and Chinese-RoBERTa-wwm), we use the output feature of the (CLS) token as the document embedding, followed by a feedforward layer to derive the final prediction. For the pretraining tasks in BERT and RoBERTa, Chinese text is segmented according to the granularity of characters, and subwords represent independent Chinese characters. Chinese-BERT-wwm and Chinese-RoBERTa-wwm use the whole word masking strategy for pretraining.

For TextGCN-Word, we follow the TextGCN protocols (Yao, Mao & Luo, 2019) to preprocess the data. We construct a heterogeneous graph with words and documents as nodes for the dataset and use Chinese-BERT-wwm for document embedding. The weights of edges between nodes are defined to be the same as those in TextGCN (Yao, Mao & Luo, 2019). GCN is used as the classifier.

For TextGCN-Character, we construct a heterogeneous graph with characters and documents as nodes for the dataset and use BERT-base to obtain document embeddings. The weights of edges between nodes are defined in the same way as those of TextGCN (Yao, Mao & Luo, 2019). GCN is used as the classifier.

### Evaluation indices

The accuracy (ACC), macroaveraged precision (P), macroaveraged recall (R), and macroaveraged F1 (F1) metrics are used as evaluation indices in this experiment. In particular, a weighted macroaveraging strategy is used for the iFlytek dataset because of its class imbalance. For binary and multiclass data, weighted recall is equivalent to accuracy.

### Datasets

We conduct experiments on three widely used Chinese benchmark corpora, including ChnSentiCorp (Cui et al., 2021), Toutiao-S and iFlytek (Xu et al., 2020). The statistics of the preprocessed datasets are summarized in Table 1.

**Table 1:** Summary statistics of datasets: the number of training sets, validation sets and test sets of three kinds of datasets, the number of categories, the average length, the number of nodes and the number of edges of each dataset.

Dataset	Num of samples Training/Validation/Test/Vocabulary	Num of categories	Average length	Num of nodes	Num of edges (million) PMI* of words
IFLYTEK	12,133/2,599/0/250,862	119	120	265,594	26.096
ChnSentiCorp	9,600/1,200/1,200/58,932	2	109	70,932	6.126
Toutiao-S	15,000/2,500/2,500/36,246	5	25	56,246	1.761

The ChnSentiCorp dataset was collected by Songbo Tan from the Chinese sentiment mining hotel review *corpus* of Ctrip, and it contains four subsets. In this article, three subsets of the balanced *corpus*, including online shopping reviews of hotels, laptops, and books, are selected and divided into two types of texts: texts with positive and negative sentiments.

The Toutiao dataset comes from the Chinese news headline classification *corpus* of the Toutiao client and contains a total of 380,000 pieces of data divided into 15 categories. The Toutiao-S dataset, which is a subset of Toutiao, contains news headlines in five categories: culture, finance, education, international news, and games. The text is relatively short, and each category contains 4,000 pieces of data.

The iFlytek dataset was derived from the open source *corpus* of iFlytek. It is long text annotation data about APP application descriptions and includes 119 types of application topics related to daily life. The accuracy rate in the experiment was calculated only on the validation set, since the test set of iFlytek does not contain category labels.

### Parameter settings

The experimental hardware configuration is as follows. The graphics card is an RTX 3090 with 24 GB of video memory. The CPU is an AMD EPYC 7601 with 64 GB of memory. The development language is Python 3.9, the development platform is PyTorch 1.10.1, and the development tool is PyCharm. Due to differences in text length, number of categories, dataset size, and limited hardware conditions for different datasets, different parameter values are set for each dataset. The specific settings are shown in Table 2.

**Table 2:** Parameter settings.

Dataset	MaxLen	Window size	Batch size
ChnSentiCorp	256	20	32
Toutiao-S	128	20	64
iFlytek	256	30	32

**Figure 2:**
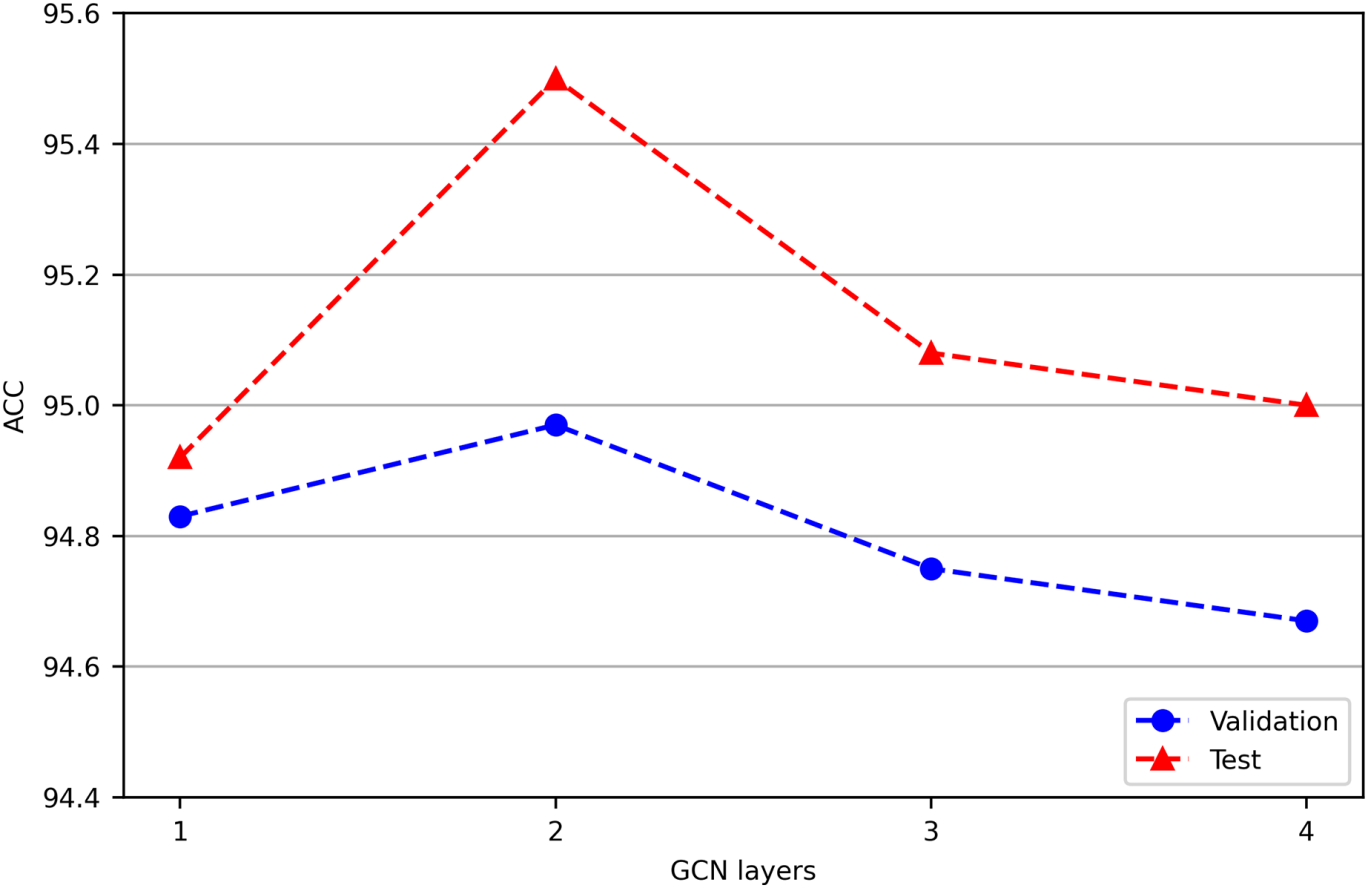
Performance comparison of GCN models with different layers on ChnSentiCorp. We run all models three times and report the mean result on validation and test sets.

In our preliminary experiment, we jointly train Chinese-BERT-wwm and GCN with different layers on the ChnSentiCorp validation and test datasets, and the results are shown in Fig. 2. We find that a two-layer GCN performs better than a one-layer GCN, while adding more layers does not yield improved performance. This is similar to the TextGCN results (Yao, Mao & Luo, 2019). From a spatial perspective, a one-layer GCN is equivalent to aggregating first-order neighbor node information, while multilayer stacking is equivalent to continuously increasing the aggregation radius. When the number of GCN layers reaches a certain depth, a node in the graph may aggregate the information of the whole graph. This situation leads to overfitting for the node classification task, and the classification effect declines instead. Increasing the number of GCN layers also increases the time complexity of the model. Therefore, the GCN used in our experiment is a two-layer GCN model.

We tune the learning rates and initialize them to 2e−5 for the GCN module and 2e−6 for the fine-tuned BERTology-series module. We tune the other parameters and set the dropout rate as 0.5. For BERTology models, the number of epochs is controlled to 10, while that for the other models is 100. The remaining variables are set to their default values.

The parameter $C$ in Eq. (1) controls whether to add edges between words on short text corpora. Only when the cosine similarity between two words is greater than $C$, we add an edge between them and use cosine similarity to represent the edge weight. We calculate the cosine similarity between words in the vocabulary of Toutiao-S and found that the median is about 0.8, and the upper 1/4 quartile is about 0.85. When $C = 0.85$, the classification accuracy and training speed are both good. Therefore, $C$ in Eq. (1) is set to 0.85 in our experiments on Toutiao-S.

**Figure 3:**
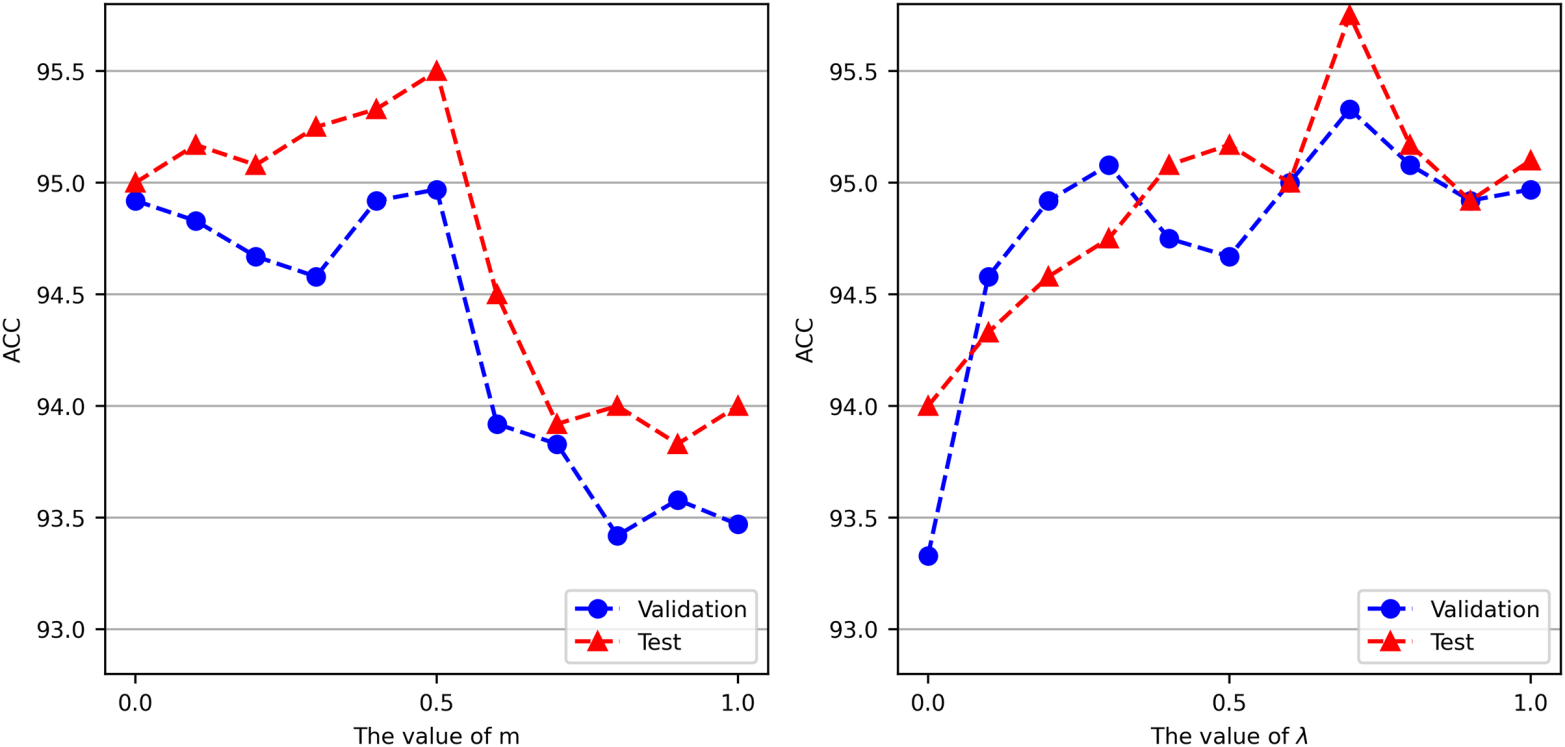
Accuracy of Chinese-BERT-wwm-GCN when varying parameters m and λ on ChnSentiCorp.

The parameter $m$ in Eq. (7) controls the tradeoff between training GCN and Chinese-BERT-wwm. To explore the optimal value of $m$, we set different $m$ values for conducting experiments on the ChnSentiCorp validation and test datasets. For each $m$ value, we conduct three experiments and report the mean accuracy. The left side of Fig. 3 shows the accuracy achieved by Chinese-BERT-wwm-GCN with different $m$ values. On ChnSentiCorp, as $m$ gradually increases, the accuracy first increases and then decreases. When $m$ is 0.5, Chinese-BERT-wwm-GCN attains its best performance, performing slightly better than using only Chinese-BERT-wwm ( $m = 0$) or GCN ( $m = 1$) for prediction. This shows that Chinese-BERT-wwm-GCN can make full use of the advantages of large-scale pretrained language models and graph-based methods. The optimal value of $m$ can be different for different tasks. For the convenience of comparison, $m$ in Eq. (7) is uniformly set to 0.5 in our experiments.

The parameter $\lambda$ in Eq. (14) controls the proportions of the cross-entropy and hinge losses. To explore the optimal value of $\lambda$, we set different $\lambda$ values for experiments conducted on the ChnSentiCorp validation and test datasets when $m = 0.5$. For each value of $\lambda$, we conduct three experiments and report the mean accuracy. The right side of Fig. 3 shows the accuracy of Chinese-BERT-wwm-GCN obtained with different $\lambda$ parameters. On ChnSentiCorp, as $\lambda$ increases, that is, as the proportion of the cross-entropy loss increases, the accuracy exhibits obvious fluctuations. The cross-entropy loss only depends on the logarithm of the probability that the model predicts the correct class, but it has a high learning rate. However, the hinge loss not only needs to be classified correctly but also requires that the loss be 0 when the distance between the incorrectly classified category and the correctly classified category is sufficiently large. Therefore, the hinge loss function has higher requirements for model learning. Our experiments show that combining two loss functions for model training yields better performance than using only the cross-entropy loss ( $\lambda$ = 1) or the hinge loss ( $\lambda$ = 0), and when $\lambda = 0.7$, Chinese-BERT-wwm-GCN attains its best performance. Therefore, λ in Eq. (14) is set to 0.7 in our experiments.

## Experimental results and discussion

We conduct experiments and comparative analyses from three aspects: comparative experiments, ablation experiments and experiments regarding the effect of the labeled data size. Among them, Chinese-BERTology-wwm-GCN represents the original model formed by jointly training the Chinese-BERTology-wwm and GCN modules for Chinese text classification. For Chinese-BERTology-wwm-GCN, we construct a heterogeneous graph with words and documents as nodes for the dataset and use Chinese-BERTology-wwm for text embedding. The weights of edges between nodes are defined in the same manner as those in TextGCN (Yao, Mao & Luo, 2019). The loss function is the cross-entropy loss. Chinese-BERTology-wwm-GCN-L introduces the strategy of fusing the cross-entropy and hinge losses based on Chinese-BERTology-wwm-GCN. Chinese-BERTology-wwm-GCN-P introduces the PMI* of words based on Chinese-BERTology-wwm-GCN. Chinese-BERTology-wwm-GCN-S uses cosine similarity to represent the edge weights between words based on Chinese-BERTology-wwm-GCN. Chinese-BERTology-wwm-GCN-LP introduces both the PMI* of words and the strategy of fusing the cross-entropy and hinge losses based on Chinese-BERTology-wwm-GCN. Chinese-BERTology-wwm-GCN-LS introduces both the cosine similarity of words and the strategy of fusing the cross-entropy and hinge losses based on Chinese-BERTology-wwm-GCN.

### Comparative experiment

**Figure 4:**
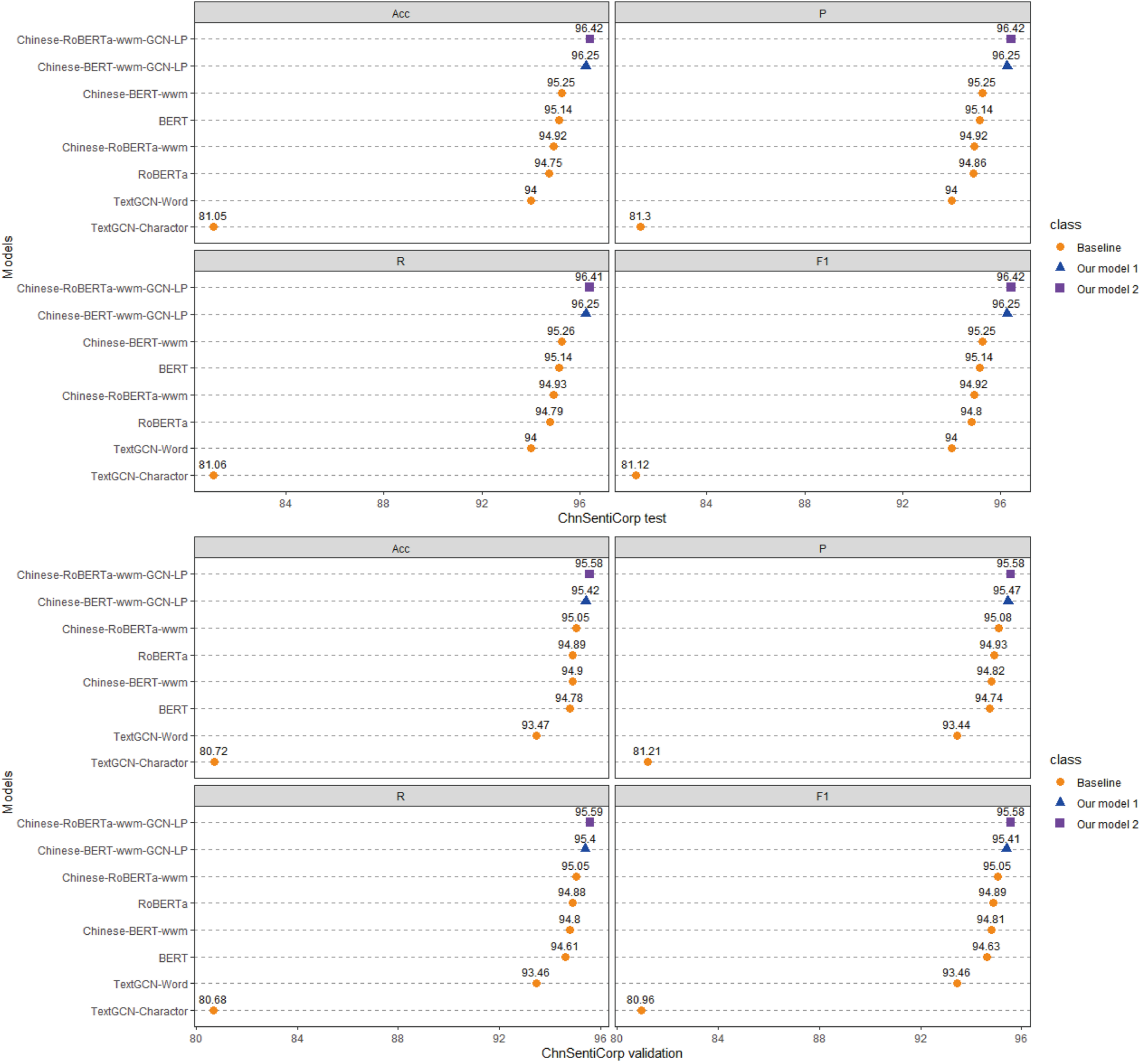
Performance comparison with baselines on ChnSentiCorp. We run all models three times and report the mean result on validation and test sets.

**Figure 5:**
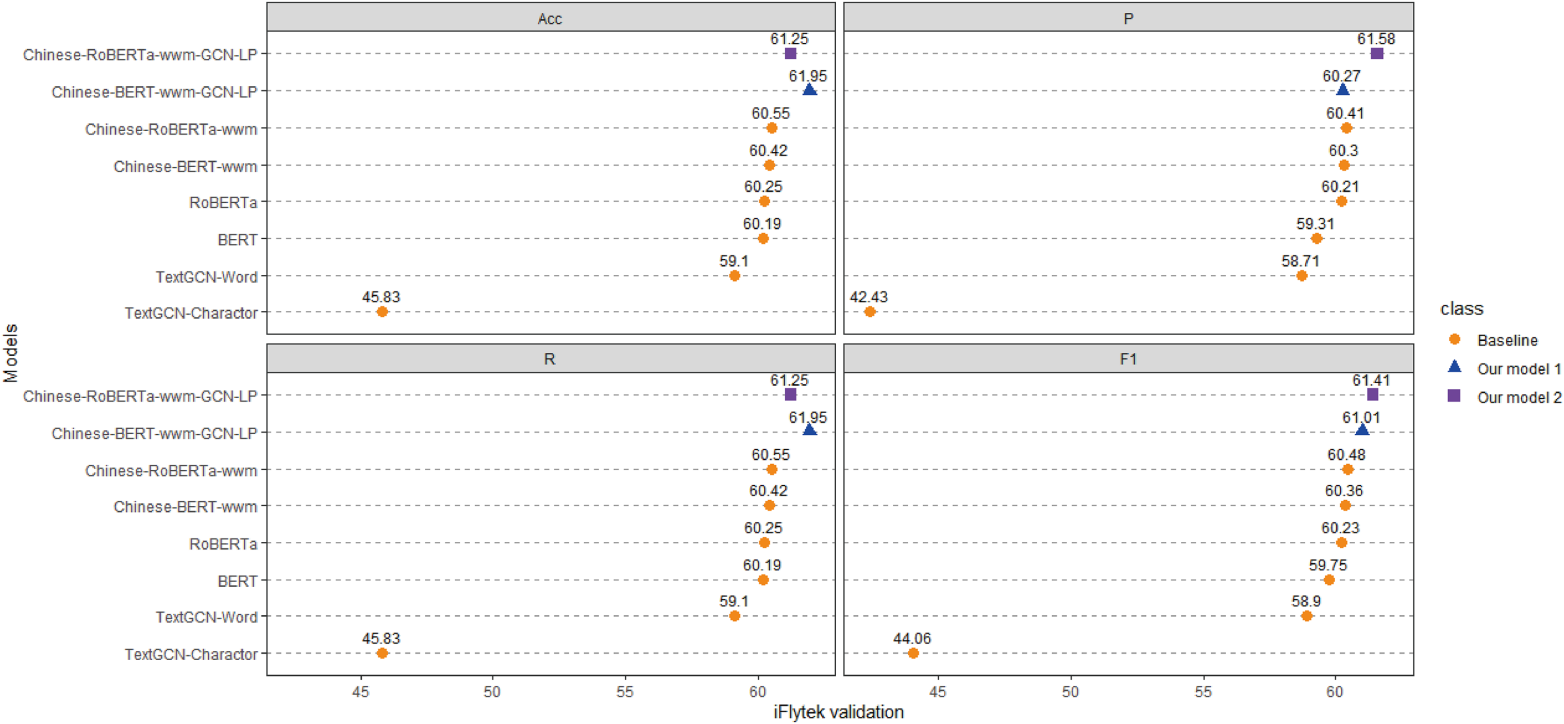
Performance comparison with baselines on iFlytek. We run all models three times and report the mean result on validation sets.

**Figure 6:**
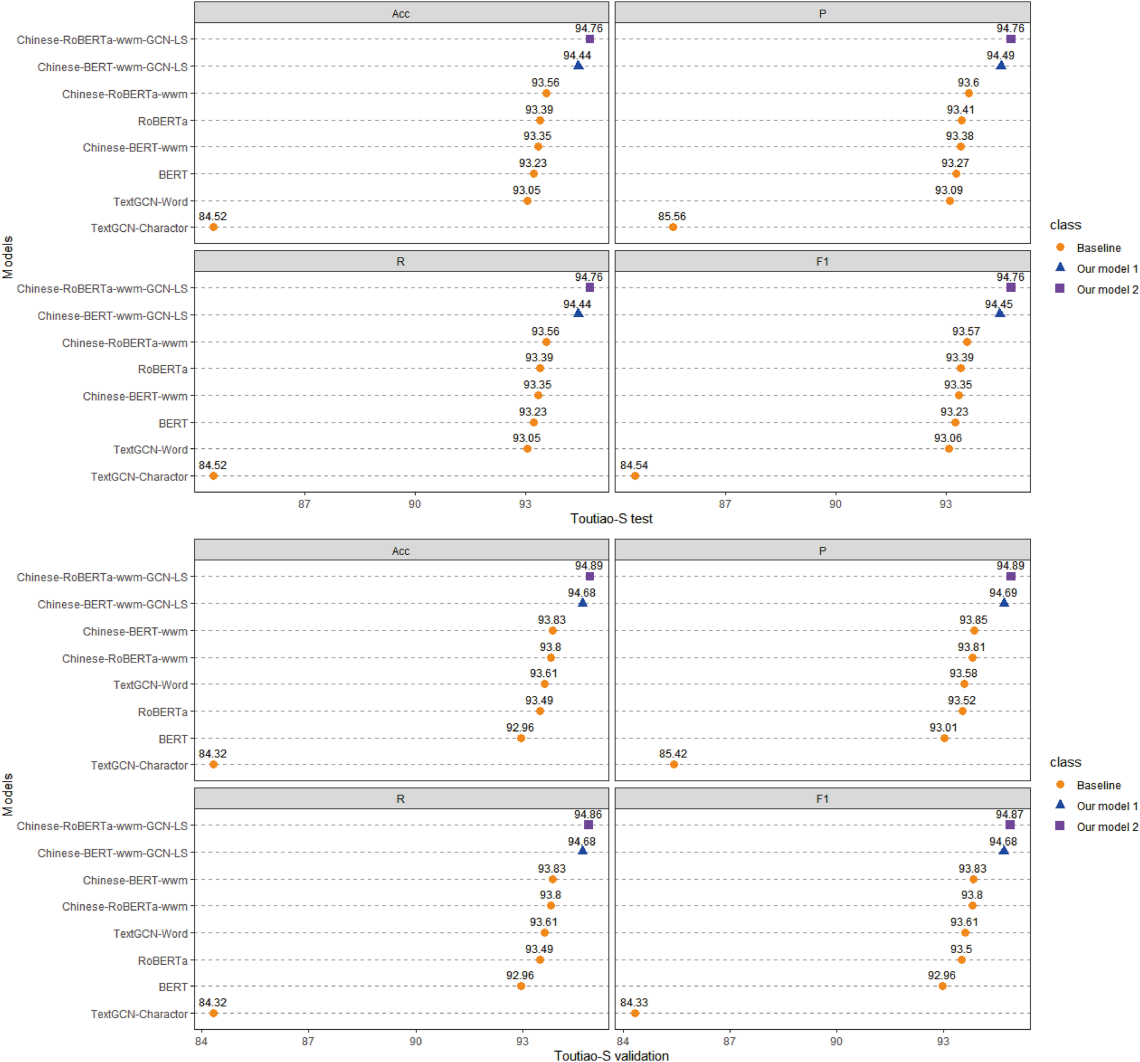
Performance comparison with baselines on Toutiao-S. We run all models three times and report the mean result on validation and test sets.

Each model is run three times on the ChnSentiCorp, Toutiao-S and iFlytek datasets, and the average ACC, P, R, and F1 values of the three runs are taken as the final results for each dataset. The experimental results are shown in Figs. 4–6. The following can be observed from the experimental results.

(1) Compared with the baseline models, our models (Chinese-BERTology-wwm-GCN-LP, Chinese-BERTology-wwm-GCN-LS) achieve the best results in terms of ACC, P, R, and F1 on the three datasets. This indicates the effectiveness of our proposed framework in Chinese text classification tasks.

(2) BERTology (BERT, RoBERTa, Chinese-BERT-wwm and Chinese-RoBERTa-wwm) models generally perform slightly better than TextGCN models (TextGCN-Word and TextGCN-Character), which is due to the great merits brought by large-scale pretraining.

(3) Compared with TextGCN-Character, the performance boost from TextGCN-Word is significant on the three datasets. Among the BERTology models, Chinese-BERT-wwm and Chinese-RoBERTa-wwm perform slightly better than BERT and RoBERTa, respectively. These results indicate that using words as the smallest granularity level is more effective than using Chinese characters in Chinese text classification tasks.

### Ablation experiment

The results of the module ablation experiment are shown in Tables 3–5. From the above experimental results, we can draw the following conclusions.

**Table 3:** Ablation experiment results on Chnsenticorp.

Dataset (Chnsenticorp)	Validation				Test			
Evaluation index (%)	Acc	*P*	R	F1	Acc	*P*	R	F1
Chinese-BERT-wwm-GCN	94.97	95.04	94.95	94.97	95.50	95.57	95.53	95.50
Chinese-BERT-wwm-GCN-L	95.33	95.42	95.31	95.33	95.75	95.75	95.75	95.75
Chinese-BERT-wwm-GCN-P	95.25	95.26	95.24	95.25	95.83	95.84	95.83	95.83
Chinese-BERT-wwm-GCN-LP	95.42	95.47	95.40	95.41	96.25	96.25	96.25	96.25
Chinese-RoBERTa-wwm-GCN	95.14	95.14	95.14	95.14	95.22	95.24	95.22	95.22
Chinese-RoBERTa-wwm-GCN-L	95.33	95.35	95.33	95.33	95.64	95.65	95.65	95.64
Chinese-RoBERTa-wwm-GCN-P	95.25	95.26	95.25	95.25	95.86	95.87	95.86	95.86
Chinese-RoBERTa-wwm-GCN-LP	95.58	95.58	95.59	95.58	96.42	96.42	96.41	96.42

**Note:**

Remove one or two modules from our model to verify the effect of each module. We run all models three times and report the mean result on validation and test sets.

**Table 4:** Ablation experiment results on iFlytek.

Dataset (iFlytek)	Validation			
Evaluation index (%)	Acc	*P*	R	F1
Chinese-BERT-wwm-GCN	60.50	59.21	60.50	59.90
Chinese-BERT-wwm-GCN-L	60.79	60.21	60.79	60.50
Chinese-BERT-wwm-GCN-P	60.91	59.83	60.91	60.37
Chinese-BERT-wwm-GCN-LP	61.95	60.27	61.95	61.01
Chinese-RoBERTa-wwm-GCN	60.79	59.19	60.79	59.98
Chinese-RoBERTa-wwm-GCN-L	61.13	60.37	61.13	60.75
Chinese-RoBERTa-wwm-GCN-P	61.09	60.30	61.09	60.69
Chinese-RoBERTa-wwm-GCN-LP	61.25	61.58	61.25	61.41

**Note:**

Remove one or two modules from our model to verify the effect of each module. We run all models three times and report the mean result on validation and test sets.

**Table 5:** Ablation experiment results on Toutiao-S.

Dataset (Toutiao-S)	Validation				Test			
Evaluation index (%)	Acc	*P*	R	F1	Acc	*P*	R	F1
Chinese-BERT-wwm-GCN	93.89	93.91	93.89	93.89	93.37	93.39	93.37	93.38
Chinese-BERT-wwm-GCN-L	93.97	93.99	93.97	93.97	93.67	93.74	93.67	93.69
Chinese-BERT-wwm-GCN-P	93.97	94.02	93.97	93.98	93.71	93.77	93.71	93.72
Chinese-BERT-wwm-GCN-S	94.30	94.40	94.30	94.34	94.04	94.06	94.04	94.04
Chinese-BERT-wwm-GCN-LP	94.48	94.48	94.48	94.48	94.14	94.15	94.14	94.14
Chinese-BERT-wwm-GCN-LS	94.68	94.69	94.68	94.68	94.44	94.49	94.44	94.45
Chinese-RoBERTa-wwm-GCN	93.84	93.86	93.84	93.84	93.61	93.68	93.61	93.62
Chinese-RoBERTa-wwm-GCN-L	94.16	94.20	94.16	94.16	93.99	94.02	93.99	93.99
Chinese-RoBERTa-wwm-GCN-P	94.24	94.25	94.24	94.24	94.12	94.12	94.12	94.12
Chinese- RoBERTa-wwm-GCN-S	94.50	94.52	94.50	94.50	94.40	94.44	94.40	94.41
Chinese- RoBERTa-wwm-GCN-LP	94.68	94.66	94.68	94.68	94.50	94.54	94.50	94.51
Chinese- RoBERTa-wwm-GCN-LS	94.89	94.89	94.86	94.87	94.76	94.76	94.76	94.76

**Note:**

Remove one or two modules from our model to verify the effect of each module. We run all models three times and report the mean result on validation and test sets.

(1) For the ChnSentiCorp and iFlytek datasets, although the contributions of PMI* and integrating the two loss functions to the overall model are not exactly the same, removing either module will result in performance decrease. This indicates that introducing these two modules for long text corpora is indeed useful and they complement each other in terms of functionality.

(2) For the Toutiao-S dataset, removing any module leads to varying degrees of degradation in classification performance. Among them, the edge weights between words represented by PMI* have a slight impact on model performance, while the weights between words represented by cosine similarity can significantly improve the performance of Chinese short text classification.

From Table 1 we can see that ChnSentiCorp and iFlytek are both long text datasets containing a large number of word nodes. Text graphs are typically constructed based on the co-occurrence relations between words and documents, with long documents giving rise to a greater number of co-occurring nodes. Thus, the text graphs constructed from long texts usually have more edges than that constructed from short texts. This indicates that longer documents contain more abundant graph structural information. Although there are no direct edges between documents, the two-layer GCN allows information to propagate between documents through intermediate word nodes, which facilitates the propagation of information on the graph.

Toutiao-S is a short text dataset. Typically, short text datasets contain a relatively small number of words, and their co-occurrence relations are sparser. PMI* between words is calculated based on their co-occurrence relations. Therefore, when using PMI* to represent edge weights between words, the number of edges in the text graph is relatively small, which limits the propagation of information between nodes. GCN usually achieves better performance in tasks with richer structural relationships, which are more conducive to transductive learning. When using cosine similarity to represent the weight of edges between words, we no longer rely on their co-occurrence relations in the *corpus*. Although we only build edges between words with cosine similarity greater than 0.85 on the Toutiao-S dataset, this still further enriches the structural information of the text graph. As a result, Chinese-BERTology-wwm-GCN-E significantly improves the classification accuracy on the Toutiao-S dataset. Moreover, it complements the loss function module and further enhances the classification performance on Chinese short text.

### Effect of the labeled data size

**Figure 7:**
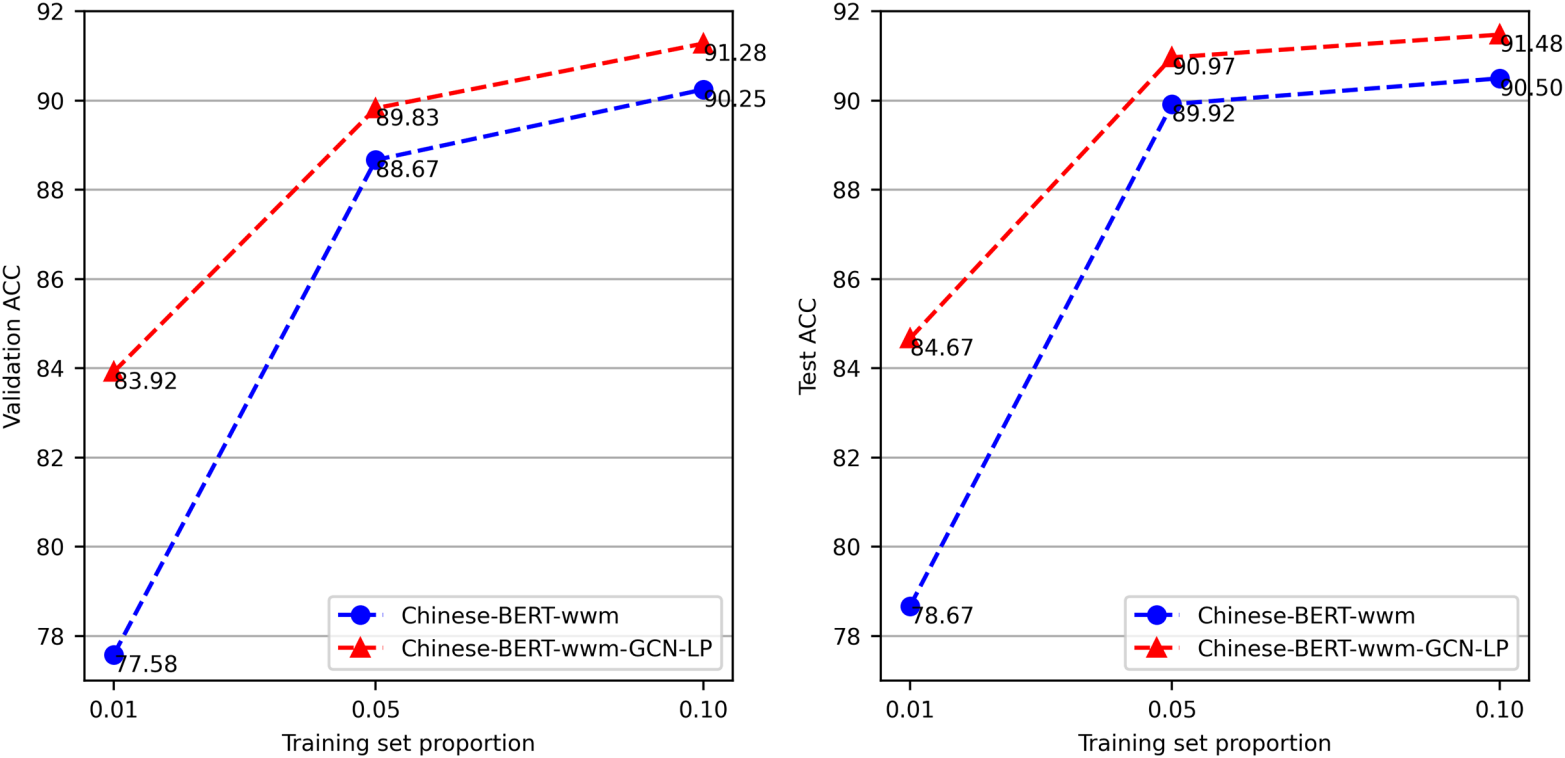
Accuracy on ChnSentiCorp by varying training data proportions.

To evaluate the effect of the size of the labeled data, we conduct tests with different training data proportions. We use the proportional sampling method to respectively extract 1%, 5%, and 10% of the data from the ChnSentiCorp training set. We perform each experiment three times and report the average accuracy attained on the test and validation sets. From the results in Fig. 7, we can see that Chinese-BERT-wwm-GCN-LP performs well on the training set with a limited number of labels. Compared with Chinese-BERT-wwm, the accuracy of Chinese-BERT-wwm-GCN-LP is significantly higher when using only 1% of the training set. As the training data quantity increases, the gap between the two methods gradually decreases.

GCN can perform well with a low label rate because heterogeneous text graphs with words and documents as nodes preserve global node relationship information, and GCN can effectively propagate document label information to the whole graph. This further verifies the advantages of our proposed method in classification tasks with low label rates.

## Conclusions and future directions

In this study, we take full advantage of large-scale pretraining and transductive learning and propose a Chinese text classification framework termed Chinese-BERTology-wwm-GCN. We build heterogeneous graphs incorporating words and documents nodes and assign different word-word edge weights for the long and short text corpora, respectively. We conduct extensive empirical studies and results analyses on three Chinese benchmark datasets through comparative experiments, ablation experiments and labeled data size experiments. Our experimental results demonstrate that utilizing the PMI* measure for words can lead to improved performance in Chinese long text classification tasks, while utilizing the cosine similarity measure for words can enrich the graph structure information of short text, thereby improving the performance of Chinese short text classification tasks. Furthermore, applying a fusion of cross-entropy and hinge losses into the Chinese-BERTology-wwm-GCN training process can further improve the performance of Chinese text classification. We expect to enhance the Chinese text classification by introducing glyph and pinyin information in future works.

## Supplemental Information

10.7717/peerj-cs.1544/supp-1 Supplemental Information 1 Datasets and codes for experiments, including ChnSentiCorp, Toutiao-S and IFLYTEK.

## Additional Information and Declarations

### Data Availability

The following information was supplied regarding data availability: The data and code are available in the Supplemental Files. The ChnSentiCorp dataset is also available at GitHub: https://github.com/ymcui/Chinese-BERT-wwm/tree/master/data/chnsenticorp. The Toutiao-S and IFLYTEK datasets are also available at GitHub: https://github.com/CLUEbenchmark/CLUE.
